# Positive and negative maternal mental health demonstrate distinct pathways to childhood depression

**DOI:** 10.1017/S0033291726103894

**Published:** 2026-05-28

**Authors:** Pei Huang, Aisleen Mariz Arellano Manahan, Marissa Y.H. Lee, Shi Yu Chan, Zhen Ming Ngoh, Michelle Z.L. Kee, Desiree Y. Phua, Anu S. Sathyapalan, Yap-Seng Chong, Peter D. Gluckman, Helen Chen, Marielle V. Fortier, Lourdes M. Daniel, Juan H. Zhou, Evelyn C. Law, Michael J. Meaney, Ai Peng Tan

**Affiliations:** 1 Institute for Human Development and Potential, Agency for Science, Technology and Research, Singapore; 2 Department of Obstetrics and Gynaecology, National University Health System, Singapore; 3 Yong Loo Lin School of Medicine, National University of Singapore, Singapore; 4 Liggins Institute, University of Auckland, New Zealand; 5 Department of Psychological Medicine, KK Women’s and Children’s Hospital, Singapore; 6 Duke-National University of Singapore, Singapore; 7 Department of Diagnostic and Interventional Imaging, KK Women’s and Children’s Hospital, Singapore; 8 Department of Child Development, KK Women’s and Children’s Hospital, Singapore; 9 Lee Kong Chian School of Medicine, Singapore; 10 Centre for Sleep and Cognition & Centre for Translational Magnetic Resonance Research, Yong Loo Lin School of Medicine, National University of Singapore, Singapore; 11 Department of Electrical and Computer Engineering, National University of Singapore, Singapore; 12 Integrative Sciences and Engineering Programme (ISEP), National University of Singapore, Singapore; 13 Department of Pediatrics, Khoo Teck Puat-National University Children’s Medical Institute, National University Health System, Singapore; 14 Douglas Mental Health University Institute, Department of Psychiatry, Faculty of Medicine, McGill University, Montreal, Canada; 15 Department of Diagnostic Imaging, National University Health System, Singapore

**Keywords:** childhood depression, executive function, language, maternal depression, positive maternal mental health

## Abstract

**Background:**

Maternal mental health strongly influences child development and depression risk. This study investigated how positive and negative dimensions of prenatal maternal mental health differentially shape childhood depressive symptoms through cognitive mediators.

**Methods:**

Participants were drawn from the *Growing Up in Singapore Towards Healthy Outcomes* (GUSTO) cohort. Of the 1198 mother–child dyads enrolled, 523 (52.6% boys) had sufficient data for the mediation analysis. Maternal mental health at 26 weeks’ gestation was assessed using a bifactor model derived from the Beck Depression Inventory-II, State–Trait Anxiety Inventory, and Edinburgh Postnatal Depression Scale. Child language ability was measured at age 2 years with the Bayley Scales of Infant Development, executive function at age 7 years with the Behaviour Rating Inventory of Executive Function, and depressive symptoms at age 9 years with the Children’s Depression Inventory-2. Serial mediation models tested hypothesized pathways.

**Results:**

Distinct mediation pathways emerged. Positive maternal mental health was associated with enhanced early language ability, which in turn was associated with fewer depressive symptoms in later childhood (*β* = −0.017, 95% CI: −0.042, −0.003). Conversely, negative maternal mental health was associated with poorer executive functioning, which in turn was associated with more depressive symptoms (*β* = 0.040, 95% CI: 0.016–0.077).

**Conclusions:**

Positive and negative maternal mental health are linked to childhood depressive symptoms through distinct neurocognitive pathways. By identifying language and executive function as specific developmental mediators, our findings point to targeted and developmentally sensitive intervention opportunities to disrupt intergenerational pathways of depression.

## Introduction

Maternal mental health profoundly affects offspring development and long-term outcomes (Koutra et al., 2013; Rogers et al., 2020), including changes in brain structure and connectivity (Adamson, Letourneau, & Lebel, 2018; Lebel et al., 2016; Qiu et al., 2015), cognitive and emotional function (Grace, Evindar, & Stewart, 2003; Koutra et al., 2013), and risk of depression in later life (Pearson et al., 2013; Weissman & Jensen, 2002). The London School of Economics estimates perinatal maternal health problems to cost a shocking 8.1 billion pounds annually, of which 72% are associated with child outcomes (Bauer, Parsonage, & Iemmi, 2014). In Singapore, mental health problems have been steadily increasing over the past decade (Harish et al., 2021) and was reported to be the largest cause of years lost to disease among children and adolescents (Epidemiology & Disease Control Division, 2019). Critically, maternal mental health is modifiable, thus representing a key intervention target to optimize child outcomes (Weissman, 2016; Weissman et al., 2015).

Although maternal mental health shapes child development, there remains a striking paucity of research examining its full spectrum. To date, research has overwhelmingly targeted negative maternal mental health, which includes psychological distress, depression, and anxiety (Gold & Marcus, 2008; Satyanarayana, Lukose, & Srinivasan, 2011), while overlooking the complementary domain of positive maternal mental health. Positive maternal mental health is not simply the absence of negative symptoms (Phua et al., 2020; World Health Organization (WHO), Victorian Health Promotion Foundation (VicHealth), & The University of Melbourne, 2004), but the combined self-perceived well-being and psychosocial functioning in mothers that improves personal competency for overcoming life challenges and flourishing in life (Henrichs & Witteveen, 2022; Norriss, 2010; Public Health Agency of Canada, 2014). Preexisting psychiatric screening measures such as the State–Trait Anxiety Inventory (STAI) (Spielberger et al., 1983) comprise positively worded items (e.g. ‘I am content’) that can be used to study and assess positive maternal mental health effects (Phua et al., 2017). This approach is further supported by a study that used positively worded items on the General Health Questionnaire for the assessment of positive maternal mental health (Hu, Stewart-Brown, Twigg, & Weich, 2007).

Positive maternal mental health represents a conceptually and functionally distinct dimension from negative maternal mental health, exerting distinct influences on child development. Independent of negative maternal mental health, positive maternal mental health predicts better language development and increased social competence in the offspring (Phua et al., 2017). Moreover, mothers who report greater positive maternal mental health confer protection against offspring psychopathology, with their children showing lower rates of developmental delays and reduced prevalence of behavioral and mental disorders in later childhood (Estinfort et al., 2022; Lähdepuro et al., 2022; Tough et al., 2008). Positive maternal mental health also attenuates the association between negative maternal mental health and offspring internalizing symptoms, suggesting a buffering effect on neurobiological stress pathways (Clayborne et al., 2022). In contrast, negative maternal mental health may engage distinct mechanisms such as dysregulated HPA-axis activity and pro-inflammatory processes (Dickens & Pawluski, 2018; Weinstock, 2005) that independently shape brain development and risk trajectories (Gustafsson et al., 2018; V. X. Han et al., 2021). It is therefore important to disentangle the specific biological, developmental, and psychosocial pathways through which each dimension independently contributes to child mental health outcomes (Keyes, 2007; Lähdepuro et al., 2022; Suldo & Shaffer, 2008; Weich et al., 2011).

We propose targeting language development and executive function as two mechanistic pathways through which both positive and negative maternal mental health may shape risk for childhood depression (M. X. Han et al., 2024; Sandman, Buss, Head, & Davis, 2015; Sawyer, Zunszain, Dazzan, & Pariante, 2019). Both language skills and executive function have been linked to maternal mental health (Ahun et al., 2017; Buss, Davis, Hobel, & Sandman, 2011; Park, Brain, Grunau, Diamond, & Oberlander, 2018; Wang & Dix, 2017), and deficits in these domains, in turn, predict greater depressive symptoms and socio-emotional problems in adolescents (G. Han et al., 2016; Irwin, Carter, & Briggs-Gowan, 2002; Kavanaugh et al., 2020). Moreover, both language and executive function critically underpin academic competence (Hebert-Myers, Guttentag, Swank, Smith, & Landry, 2006; Pearson et al., 2016), which is predictive of later depressive symptoms (Cole, Martin, & Powers, 1997; Cole, Martin, Powers, & Truglio, 1996). Positive maternal mental health is associated with increased language competencies (Phua et al., 2017, 2020; Tough et al., 2008), possibly mediated by more responsive parent–child interactions and enriched linguistic environments (Phua et al, 2020). In contrast, negative maternal mental health has been linked to delays in language acquisition (A. D. Cox, Puckering, Pound, & Mills, 1988; Göker, Eser, & Yilmaz, 2019; Letourneau, Tramonte, & Willms, 2013; Quevedo et al., 2012), potentially due to reduced verbal engagement and increased emotional unavailability. Children and adolescents with language impairments also exhibit more depressive symptoms than healthy children (St Clair, Skeen, Marlow, & Tomlinson, 2019; van den Bedem et al., 2018; Wadman, Botting, Durkin, & Conti-Ramsden, 2011) and are at an increased risk of future depressive symptoms (Botting et al., 2016; Herman et al., 2016). Furthermore, toddlers with impaired expressive language have 17 times higher depression and withdrawal ratings than their healthy counterparts (Irwin et al., 2002). Similarly, executive function in childhood also mediates the association between perinatal maternal mental health and offspring academic achievement at 16 years (Pearson et al., 2016). This pathway may reflect the impact of maternal depression on the early development of cognitive control processes, which are crucial for emotion regulation and adaptive coping (Chae et al., 2020; Priel, Zeev-Wolf, Djalovski, & Feldman, 2020). Children exposed to maternal depressive symptoms may receive less consistent scaffolding due to reduced parental sensitivity and less structured caregiving environments. By examining language skills and executive function as both parallel and serial pathways, we aim to unravel how different dimensions of maternal mental health converge and diverge in shaping offspring depression risk.

The current literature reveals two critical gaps in our understanding of how maternal mental health influences childhood depression. First, few studies have employed a comprehensive, longitudinal framework that integrates maternal mental health, intermediary cognitive factors, and child outcomes within a single unified model. Most studies are either cross-sectional or focus only on part of the pathway: either the link between maternal mental health and the mediating factor (Ahun et al., 2017; Pearson et al., 2013), or the mediator and child outcomes (Cole et al., 1996; Waszczuk, Zavos, Gregory, & Eley, 2014). Second, while negative maternal mental health has been widely studied, the influence of positive maternal mental health remains relatively underexplored. A nuanced understanding of maternal influences on child depression should require examining both conceptually distinct dimensions.

**Figure 1.**
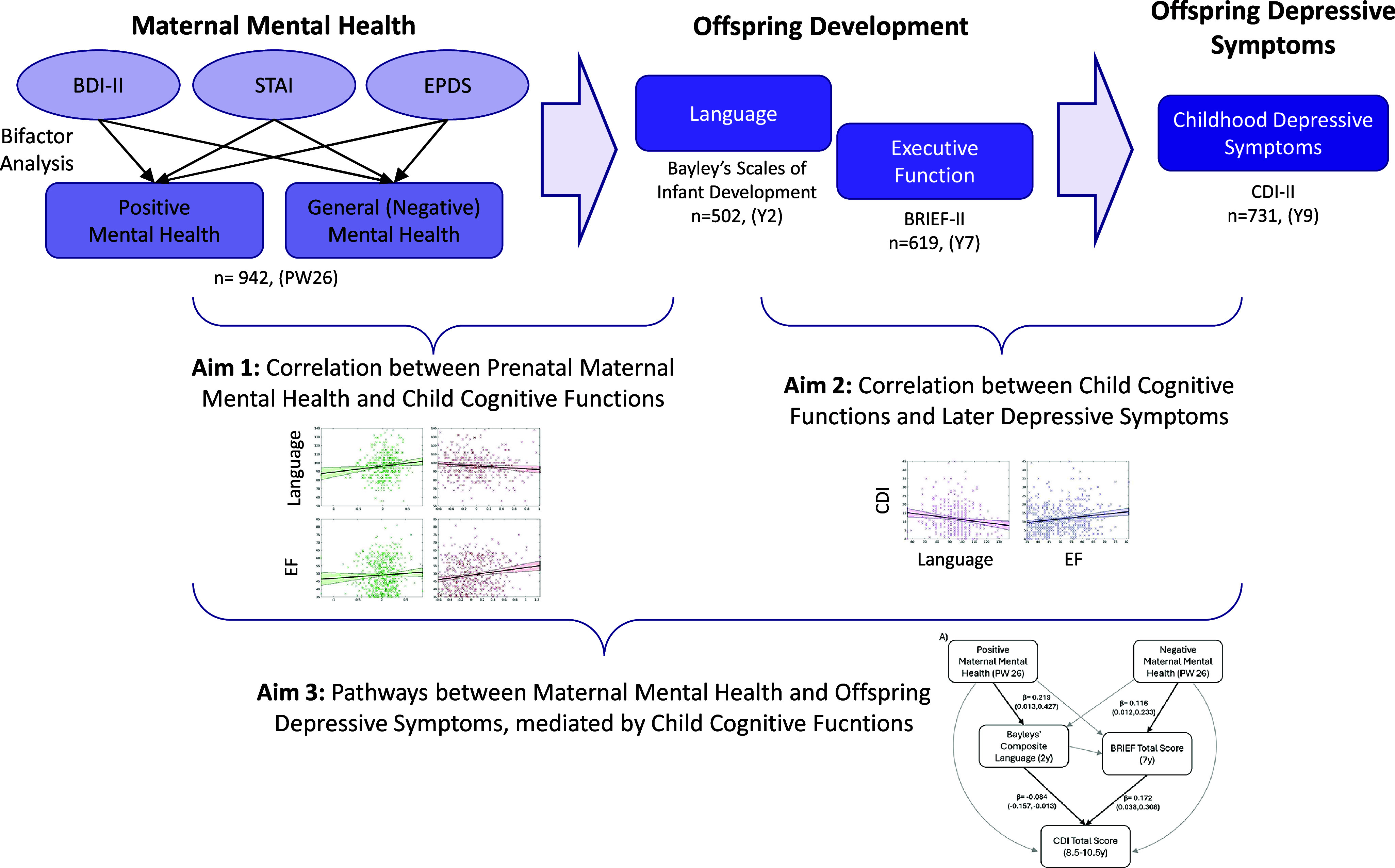
Bifactor analysis was performed on the items from three maternal mental health questionnaires (Beck Depression Inventory [BDI], State Trait Anxiety Index [STAI], and Edinburgh Postnatal Depression Scale [EPDS]) to extract a positive mental health factor and a general (negative) mental health factor. Offspring development was indexed using language and executive function, measured using the Bayley’s Scales of Infant Development and Behavioural Rating Inventory of Executive Function, 2nd edition (BRIEF-2). Offspring depressive symptoms were measured using the Child Depression Inventory 2 (CDI-II). The number of participants that completed the measure and the time of administration of the measure are indicated below the respective measures. *Note:* PW, pregnancy week; Y, year.

To address these knowledge gaps, we utilized the Growing Up in Singapore Towards Healthy Outcomes (GUSTO) birth cohort to investigate the intergenerational pathways linking maternal mental health to childhood depressive symptoms. Our analysis incorporated both positive and negative dimensions of maternal mental health, as well as two key cognitive mediators (language development and executive function) that are known to support emotional and psychological resilience in children. The study design is presented in Figure 1. We first examined the correlation between prenatal maternal mental health and offspring outcomes in language and executive function. Next, we examined how language and executive function were associated with depressive symptoms in childhood. Finally, we employed a serial mediation model to test the hypothesized pathways of intergenerational transmission. In this model, positive and negative maternal mental health were treated as independent predictors, language and executive function were modeled as sequential mediators, and childhood depressive symptoms served as the primary outcome. This approach enabled us to disentangle the potentially distinct mechanisms by which positive and negative maternal mental health influences children’s long-term emotional outcomes. We hypothesized that both language and executive function would serve as mediators for intergenerational transmission of maternal mental health, with differential pathways of influence for positive and negative maternal mental health.

## Methods

### Participants

Participants for this study were recruited from Growing Up in Singapore Towards Healthy Outcomes (GUSTO), a longitudinal, population-based birth cohort. Pregnant women aged 18 years or older were recruited at their first trimester of pregnancy from two main public hospitals in Singapore between June 2009 and December 2010 (Soh et al., 2014). Mother–child dyads were followed throughout pregnancy and beyond. This study followed the Strengthening the Reporting of Observational Studies in Epidemiology (STROBE) reporting guidelines for cohort studies (Von Elm et al., 2007). To minimize potential birth complication effects, we only included children with gestational age at birth ≥34 weeks, birth weight ≥ 2000 g, and a 5-minute Apgar score ≥ 8 in our analysis. Children with a neuropsychiatric diagnosis were also excluded. All mother–child dyads utilized in this study were healthy with no pre-identified neurodevelopmental problems. Informed consent was obtained from all participants in the study.

### Maternal mental health

Prenatal maternal mental health measures were collected during week 26 of the pregnancy. Parents completed three mental health questionnaires.

The Beck Depression Inventory, Second Edition (BDI) (Beck, Steer, & Brown, 1996) is a list of 21 items describing common depressive symptoms. Each item contains four or seven statements describing the varying severity of a common depressive symptom. Participants selected the statement that best described how they felt for the past 2 weeks. The EPDS (J. L. Cox, Holden, & Sagovsky, 1987) has 10 items of depressive symptoms, and participants indicated how much each item described how they were feeling for the past 7 days on a 4-point Likert scale. The STAI (Spielberger et al., 1983) consisted of 40 items associated with the presence or absence of anxiety. For the first 20 items, participants responded to how much each item described how they felt right now on a 4-point Likert scale (a measure of state anxiety); for the next 20 items, they responded to how much the item described how they generally felt (a measure of trait anxiety). Following prior work by Desiree et al. (Phua et al., 2017), we applied a bifactor model to the pooled items from the three maternal mental health questionnaires (BDI, STAI, and EPDS) and identified two orthogonal latent constructs: a specific positive mental health factor and a general negative mental health factor.

These factor scores were used as our primary measures of positive and negative maternal mental health, respectively. All items contribute to the negative factor, while a specific subset of positively phrased items loaded onto the positive factor (Supplementary Material S1). Positive and negative maternal mental health were not significantly correlated with each other (*r* = −0.029, 95% CI: [−0.092, 0.035], *p* = 0.379). This finding is consistent with previous studies (Phua et al., 2017) and supports the exploration of positive and negative maternal mental health as distinct constructs.

### Language

At 24 months, children’s developmental functioning was assessed using the Bayley Scales of Infant and Toddler Development, Third Edition (Bayley-III) (Bayley, 2006). This standardized instrument evaluates multiple key domains of early development. We focused on the language domain and derived a composite language score by averaging the receptive and expressive communication subscale scores.

### Executive function

Behavior Rating Inventory of Executive Function, 2nd Edition (BRIEF-2; parent-report form) (Gioia, Isquith, Guy, & Kenworthy, 2015) was administered at age 7 years (*n* = 620). The BRIEF-2 is a standardized questionnaire comprising 63 items that evaluate everyday behaviors associated with executive functioning. These items are organized into nine clinical scales and three broader indices. For the present study, we used the Global Executive Composite (GEC) score, a validated summary score that provides an overall index of executive function in daily life.

### Childhood depression

Children completed the Children’s Depression Inventory 2 (CDI-II) (Kovacs, 2011), a modified version of the BDI for children. This evaluation was carried out once per child, at either their year 8.5, year 9, or year 10 visit. The CDI-II is stable across ages, applicable for both depressed and normal children (Bae, 2012; Gomez, Vance, & Gomez, 2012) and has good test–retest reliability and high internal consistency (COSTELLO & ANGOLD, 1988). The CDI-II consists of 28 questions scored on a 3-point Likert scale. A total score of 20 or above (13.4% in our cohort) indicates clinical levels of depressive symptoms. In this study, the total CDI-II score, summed across all responses, was used as the primary outcome measure of child depressive symptoms.

### Correlation analysis

We conducted Pearson’s correlation analyses between positive and negative dimensions of prenatal maternal mental health and our candidate mediators (language and executive function). We also examined correlations between each mediator and later childhood depressive symptoms.

### Serial mediation analysis

To test the hypothesized pathways linking maternal mental health to childhood depression, we carried out mediation analysis using the Lavaan Toolbox (Rosseel, 2012) in R. The model specified language ability at age 2 years as the first mediator, executive function at age 7 years as the second mediator, and depressive symptoms at age 9 years as the outcome. Both positive and negative prenatal maternal mental health scores were entered as independent predictors. Child sex, ethnicity, maternal education level, and average monthly household income were included as covariates of no interest. Participants missing data for maternal education level and average monthly household income were estimated using mean imputation. Indirect effects were estimated using bias-corrected bootstrapping with 10,000 iterations. We included all participants with at least four out of five primary variables available and missing data were handled using Full Information Maximum Likelihood (FIML) estimation within the SEM function itself.

### Sensitivity analysis

To ensure the accuracy and robustness of our serial mediation analysis, we carried out three different sensitivity analyses. First, we estimated the same model without covariates to ensure that the findings were not driven by the covariates. Second, we estimated the same model using only participants with complete data. This estimation ensures that the findings were not due to imputation. Lastly, we tested the sensitivity of the model to sample perturbation. The mediation model was reestimated in randomly drawn subsamples comprising 10–90% of the full dataset. For each sampling proportion, 10 independent subsamples were randomly generated (beginning at 90%, followed by 80%, and iteratively down to 10%).

## Results

### Cohort profile

Participant demographic characteristics are presented in Table 1. Demographic data were collected at the time of recruitment. Comparisons between the full cohort and the subset included in the mediation analyses revealed no significant differences across key variables, except for ethnicity. This difference reflects the study’s recruitment design, which was structured to mirror the ethnic composition of the general population in Singapore.

**Table 1.** Summary of demographics for the full cohort and mediation analysis cohort

	Full cohort			Mediation cohort			*χ*^2^	*p*-value
	*n*	Mean	*SD*	*n*	Mean	*SD*		
**Demographics**								
Sex	1198			523			0.00	1.00
Male	628	(52.4)		275	(52.6)			
Female	570	(47.6)		248	(47.4)			
Ethnicity	1198			523			**6.61**	**0.03**
Chinese	676	(56.4)		269	(51.4)			
Malay	303	(25.3)		167	(31.9)			
Indian	218	(18.2)		87	(16.6)			
Others	1	(0.1)		1	(0.2)			
Mother highest education	517			396			1.65	0.80
Primary	30	(5.8)		22	(5.6)			
Secondary/Technical	200	(38.7)		141	(35.6)			
GCE ‘A’ levels/University	287	(55.5)		233	(58.8)			
Monthly household income (SGD)	496			381			1.75	0.78
<2000	83	(16.7)		59	(15.4)			
2000–5999	274	(55.2)		200	(52.9)			
>6000	139	(28.0)		122	(32.0)			
**Maternal mental health**							**T-stat**	***p*-value**
Edinburgh Postnatal Depression Scale (PW26)	1151	7.44	4.50	523	7.35	4.48	0.38	0.70
Beck Depression Inventory 2 (PW26)	1101	8.53	6.29	523	8.74	6.12	0.64	0.52
State-Trait Anxiety Inventory (PW26)	1089	71.24	17.89	523	71.12	18.03	0.12	0.90
Negative Maternal Mental Health (PW26)	942	0.00	0.36	523	0.01	0.35	0.93	0.35
Positive Maternal Mental Health (PW26)	942	0.00	0.27	523	0.01	0.27	0.63	0.53
**Offspring measures**								
Bayley Composite Language Score (Y2)	502	95.74	14.40	334	95.44	13.49	0.31	0.76
BRIEF–2 GEC Score (Y7)	619	49.21	9.76	475	49.22	9.74	0.03	0.97
CDI-II Total Score (Y9)	731	11.25	7.62	493	11.68	7.58	0.98	0.32

Numbers in brackets indicate the number of subjects as a percentage of the cohort. Chi-square test was used to test for significant differences between the two cohorts for categorical variables, and t test was used to test for significant differences for continuous variables.

*Note:* BRIEF-2, Behaviour Rating Inventory for Executive Function, 2nd edition; GEC, Global Executive Composite; CDI-II, Children’s Depression Inventory 2.

### Correlation between prenatal maternal mental health and child cognitive functions

Positive maternal mental health was significantly associated with better language outcomes at age 2 years (*r* = 0.130, 95% CI: 0.031–0.225, *p* < 0.05), suggesting positive maternal mental health significantly influences early language abilities. In terms of executive function, higher negative maternal mental health was significantly associated with greater executive dysfunction at age 7 years (*r* = 0.176, 95% CI: 0.089–0.259, *p* < 0.05), whereas positive maternal mental health was not significantly associated with executive function performance. Neither positive nor negative maternal mental health showed significant direct associations with child depressive symptoms at age 9 years. These findings suggest that positive maternal mental health may shape early language skills, while negative maternal mental health exerts a stronger influence on later executive functioning – potentially contributing indirectly to later emotional outcomes. Full results from the correlation analyses are presented in Table 2, and corresponding scatterplots are shown in Supplementary Material S2.

**Table 2.** Correlation of study variables with positive and negative maternal mental health

	Positive maternal		Negative maternal	
	mental health (PW 26)		mental health (PW 26)	
Positive maternal mental health (PW 26)			−0.029	(−0.092, 0.035)
Negative maternal mental health (PW 26)	−0.029	(−0.092, 0.035)		
Bayley composite language score (Y2)	0.13	(0.031, 0.225)*	−0.103	(−0.200, −0.005)^+^
BRIEF–2 GEC score (Y7)	0.057	(−0.031, 0.144)	0.176	(0.089, 0.259)*
CDI-II Total score (Y9)	−0.021	(−0.102, 0.060)	0.08	(−0.002, 0.160)

*Note:* ^+^indicates *p* < 0.05, *indicates *p* < 0.007 (Bonferroni correction for multiple comparisons). Values in brackets indicate 95% CI. PW, pregnancy week; Y, year.

To validate these associations, we replicated our analyses in an independent sample of mother–child dyads from the Singapore Preconception Study of Long-Term Maternal and Child Outcomes (S-PRESTO) (Loo et al., 2021) cohort. Demographic characteristics of the S-PRESTO sample and detailed methodology are provided in Supplementary Material S3. Consistent with our primary findings, positive maternal mental health was significantly associated with better language outcomes at age 4 years, as measured by the Peabody Picture Vocabulary Test, Fifth Edition (PPVT-5) (Dunn, 2019) (*r* = 0.131, 95% CI: 0.008–0.249, *p* = 0.004). Similarly, higher negative maternal mental health was significantly associated with greater executive dysfunction, assessed using the Behaviour Rating Inventory of Executive Function–Preschool Version (BRIEF-P) (Sherman & Brooks, 2010) (*r* = 0.310, 95% CI: 0.172–0.437, *p* < 0.001). Although different assessment tools were used due to the absence of Bayley and BRIEF-2 data in S-PRESTO, the replicated associations across cohorts and instruments underscore the robustness of the observed links between maternal mental health, early language development, and executive functioning.

### Correlation between child cognitive functions and later depressive symptoms

**Figure 2.**
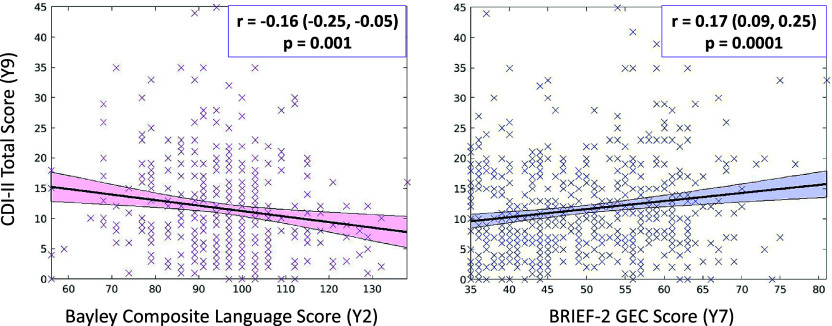
Scatterplots showing the correlation between CDI-II total score (Y9) and Bayley’s composite language score at 2 years and BRIEF-2 total score at 7 years. Correlation values, 95% CI, and *p*-values are included in the inserts.

Children’s depressive symptoms, assessed between ages 8.5 and 10 years, were significantly associated with both early language abilities and executive function. Specifically, higher depressive symptoms were correlated with lower composite language scores at age 2 years (*r* = −0.16, 95% CI: −0.252 to −0.057, *p* = 0.002) and with greater executive dysfunction, as indexed by higher BRIEF Global Executive Composite (GEC) scores at age 7 years (*r* = 0.17, 95% CI: 0.085–0.248, *p* < 0.001) (Figure 2). Visual inspection of scatterplots confirmed that these associations were continuous and not driven by outliers or extreme values. These findings align with emerging developmental models suggesting that early cognitive capacities play a foundational role in shaping emotional health. Impairments in these cognitive domains may compromise a child’s ability to navigate social and academic demands, elevating the risk of depressive symptoms in later childhood.

### Meditation pathway analysis

**Figure 3.**
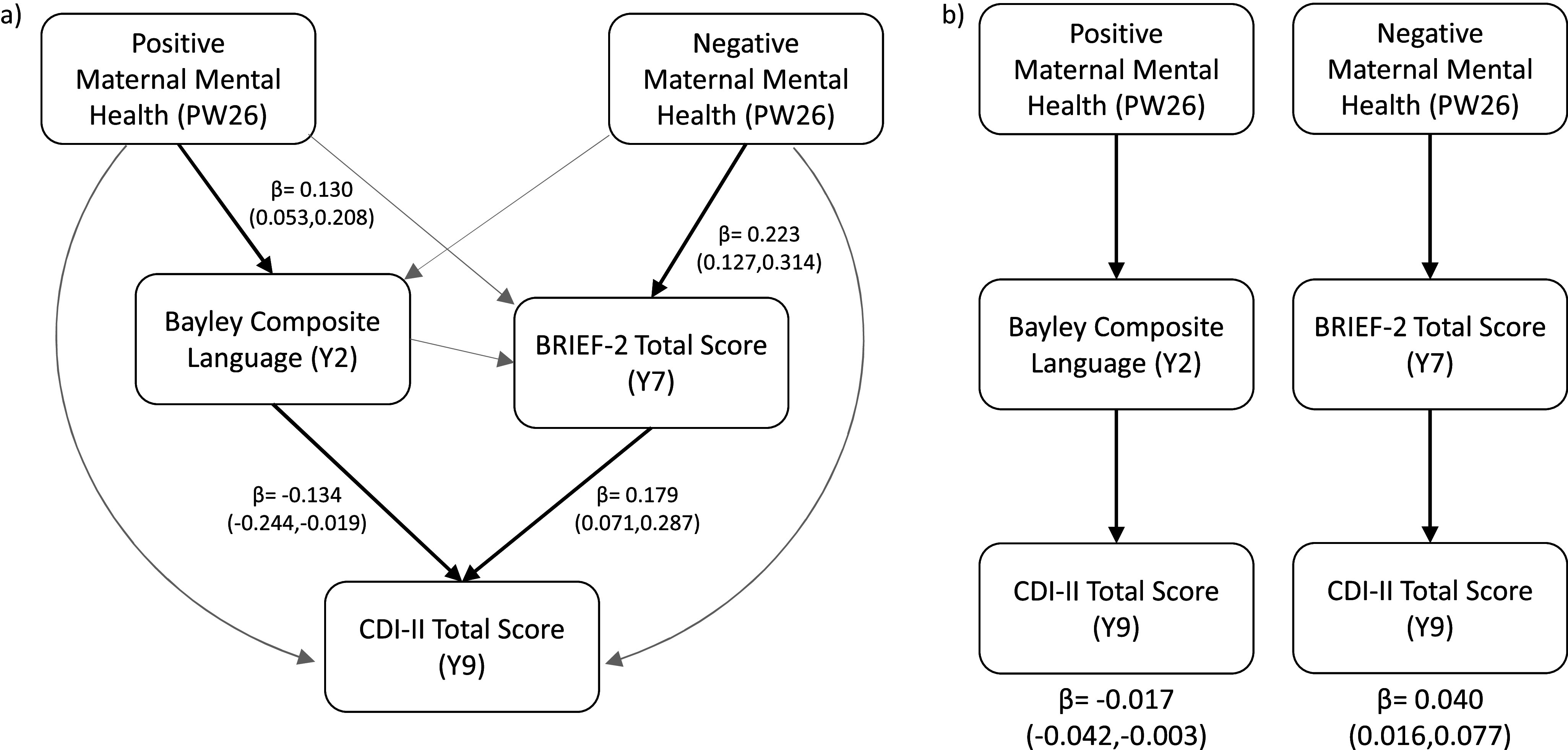
(a) A schematic illustration of the full serial mediation model (*n* = 523) employed in this study. Lines in light gray indicate relations that are not significant in regression analysis, while bold, black arrows indicate significant relations, with the beta values and confidence intervals reported next to them. (b) Two mediated pathways were significant in our mediation model and highlighted here. The beta values and confidence intervals for each pathway are reported below the figure. Full regression values and mediation pathways are presented in Supplementary Tables 2 and 3.

Language and executive function were selected as candidate mediators based on their established associations with both maternal mental health and childhood depressive symptoms. We employed a serial mediation framework (*n* = 523) to investigate how positive and negative dimensions of prenatal maternal mental health influence child depressive symptoms through intermediate cognitive processes, specifically language and executive function (Figure 3, Panel a). The analysis identified two distinct and statistically significant indirect pathways (Figure 3, Panel b). First, higher positive maternal mental health was associated with enhanced language development at age 2 years, which in turn predicted lower depressive symptoms (*β* = −0.017, 95% CI: −0.042 to −0.003). Second, higher negative maternal mental health was associated with poorer executive function at age 7 years, which in turn predicted elevated depressive symptoms (*β* = 0.040, 95% CI: 0.016–0.077). These effects were domain-specific: the protective influence of positive maternal mental health operated through early language competence, whereas the risk associated with negative maternal mental health was conveyed through disruptions in executive functioning. No other indirect pathways were statistically significant. Full regression coefficients and mediation results are presented in Supplementary Material S4.

### Sensitivity analysis

Our sensitivity analysis is presented in Supplementary Material S5. By excluding covariates and incomplete data independently, we demonstrate that our findings are robust and reliable. Further, sensitivity analysis using subsets with decreasing sample sizes shows that our findings were stable to sample size perturbations and the effects only vanished when <50% of the cohort was used.

## Discussion

Our study provides novel evidence for distinct neurodevelopmental pathways linking positive and negative dimensions of prenatal maternal mental health to the emergence of depressive symptoms in offspring. Drawing on longitudinal data from a large, well-characterized Asian birth cohort and replicating key associations in an independent sample, we demonstrate that maternal mental health during pregnancy exerts domain-specific effects on child development, operating through early language and executive function, respectively. By applying a serial mediation framework, we revealed how variations in maternal mental health shape downstream emotional outcomes via discrete cognitive mechanisms. These findings offer critical insights into the intergenerational transmission of risk and resilience, with direct relevance for the timing and targeting of early interventions. Importantly, our results underscore the value of treating positive and negative maternal mental health as partially independent constructs, rather than opposing ends of a single continuum. This critical distinction is often overlooked but essential for advancing both theoretical models and clinical applications.

We found that positive maternal mental health during pregnancy was significantly associated with enhanced language development in early childhood. This association was robust across two independent cohorts and aligned with existing evidence suggesting that maternal well-being shapes the quality of early communicative environments (Phua et al., 2017; Tough et al., 2008). Mothers experiencing greater emotional positivity may engage in more responsive and verbally enriched interactions, which supports early language acquisition. Importantly, the specific link between positive maternal mental health and language development underscores the need to conceptualize positive and negative maternal mental health as distinct constructs, rather than opposite ends of a continuum, thereby advancing a more nuanced understanding of maternal influences on child development.

In contrast, only negative maternal mental health was significantly associated with poorer executive function in middle childhood. Executive functions, such as cognitive flexibility, inhibitory control, and working memory, are critical for goal-directed behavior and emotional control (Fernandes, Wright, & Essau, 2023; Li, Liu, Yan, & Feng, 2020). Prenatal psychological distress may derail the in utero maturation of fronto-striatal and fronto-limbic circuits that underlie executive functions, possibly via immune dysregulation and epigenetic modification (Kling et al., 2023; Wu et al., 2020). Because these prenatal insults directly disrupt the fundamental building blocks of executive networks, even robust positive maternal mental health may be insufficient to fully counteract early adversity’s impact on these tightly regulated neurodevelopmental programs. Further, the optimal development of executive function may further depend on postnatal caregiving. Developmental environments rich in stimulation, consistency, and responsive scaffolding are crucial for refining executive skills through experiential learning (Bernier, Carlson, Deschênes, & Matte-Gagné, 2012; Lucassen et al., 2015).

Using a serial mediation model, we revealed two distinct and domain-specific mediation pathways linking maternal mental health to child depressive symptoms. First, the indirect association between positive maternal mental health and lower child depressive symptoms was mediated by enhanced language competency at age 2 years. This pathway suggests that positive maternal health confers protective effects on child emotional outcomes via early support of language development. While previous research has established that language impairments confer risk for internalizing problems (Botting et al., 2016; Herman et al., 2016; St Clair et al., 2019; van den Bedem et al., 2018), our use of a continuous measure highlights that language further acts as a protective factor when well developed. Although negative maternal mental health has previously been associated with delayed language development (A. D. Cox et al., 1988; Göker et al., 2019; Letourneau et al., 2013; Quevedo et al., 2012), this result was not replicated in our findings. We postulate that earlier findings may have been confounded by the absence of a bidimensional analytic framework and failing to account for the unique contribution of positive maternal mental health. In our model, the association between negative maternal mental health and child language ability was attenuated when both dimensions were examined simultaneously, suggesting that language development may be more strongly influenced by the presence of positive maternal resources than by the mere absence of negative symptoms. Second, the indirect association between negative maternal mental health and higher child depressive symptoms was mediated by executive dysfunction at age 7 years. The role of executive function as a key developmental mechanism linking negative maternal mental health to childhood depressive symptoms is consistent with a growing body of evidence demonstrating the interplay between poor maternal mental health, child executive function and internalizing problems, including depression (Buss et al., 2011; Halse, Steinsbekk, Hammar, & Wichstrøm, 2022; Hawkey, Tillman, Luby, & Barch, 2018; Wang & Dix, 2017). Executive dysfunctions may compromise a child’s capacity to manage stress and respond flexibly to emotional challenges, thus elevating vulnerability to depression. While these findings are correlational, the longitudinal structure of the data, whereby the mediators preceded depressive symptoms, strengthens the inference of potential causal pathways.

Our findings of no direct associations between maternal mental health and child depressive symptoms, in conjunction with significant indirect effects through language and executive function, are consistent with contemporary mediation theory (Preacher & Hayes, 2004) and developmental cascade models (Ettekal et al., 2020; M. X. Han et al., 2024; Masten & Cicchetti, 2010). Maternal mental health may shape foundational cognitive and self-regulatory capacities, encompassing language and executive functioning, which in turn confer resilience or vulnerability to later depressive symptoms. These mediators represent more proximal determinants of child mental health, and their inclusion may reveal mechanistic pathways even in the absence of a direct association. This pattern suggests that maternal mental health exerts its influence in a domain-specific and developmentally mediated manner, rather than through a broad, direct effect on child depression.

Together, our study findings contribute to a growing literature underscoring the influence of prenatal maternal mental health on long-term child outcomes (M. X. Han et al., 2024; Johnson & Flake, 2007; Murray et al., 2011), while advancing the field in three important ways. First, by modeling both positive and negative dimensions of maternal mental health, we show that they operate through distinct cognitive pathways rather than as a single continuum of risk. Second, the use of a serial mediation model across multiple developmental stages enhances our understanding of the timing and mechanisms through which maternal mental health shapes child functioning. Third, replicating these findings in an independent cohort using different measurement tools strengthens their generalizability and robustness.

Several limitations, however, warrant consideration. We did not assess other behavioral domains closely related to depression, such as emotional processing or reward learning (Anderson, 2007; Chau, Jarvis, Law, & Chong, 2018; Kennerley & Walton, 2011), which may confound observed pathways due to shared neural substrates. Additionally, as the study was conducted within a Singaporean birth cohort, the extent to which these findings generalize to other populations remains uncertain. Replication across more diverse cultural and genetic contexts is essential to confirm the broader applicability of these results. In addition, our analysis focused exclusively on prenatal maternal mental health. Thus, we cannot disentangle whether the observed associations reflect direct prenatal fetal programming mechanisms or prenatal depression extending into postnatal depression, leading to reduced maternal sensitivity, suboptimal parenting practices, or related environmental influences. Lastly, we did not employ recently developed instruments specifically designed to assess positive mental health, such as the Warwick-Edinburgh Mental Well-being Scale. Although the positive maternal mental health construct used here has been previously validated in published work (Phua et al., 2017), the use of more granular measures may have provided additional specificity regarding underlying dimensions of maternal well-being.

## Conclusion

In summary, our study reveals that positive and negative dimensions of maternal mental health exert distinct and opposing influences on offspring through separable neurocognitive pathways involving language and executive function. Clinically, these results underscore the need for integrated approaches that concurrently promote maternal well-being and alleviate maternal distress to optimize neurodevelopment and reduce vulnerability to later emotional disorders. Embedding such dual-focus strategies within perinatal care alongside early interventions that strengthen children’s language and executive skills may enhance resilience and interrupt the intergenerational cycle of emotional vulnerability. Future clinical trials should assess the effectiveness and scalability of these integrated prenatal–postnatal models in improving long-term outcomes for both mothers and their children.

## Supporting information

10.1017/S0033291726103894.sm001 Huang et al. supplementary material Huang et al. supplementary material

## Data Availability

The GUSTO data are not deposited into a public repository due to multisite partnership agreements and conditions for Internal Review Board approval. GUSTO data are routinely made available through submission and approval from the cohort executive committee of a data access form. Details may be obtained from the corresponding author upon reasonable request.

## Supplementary material

The supplementary material for this article can be found at http://doi.org/10.1017/S0033291726103894.
