# Supplementary material for: Positive and negative maternal mental health demonstrate distinct pathways to childhood depression
Source: Psychol Med. 2026 May 28;56:e163. doi: 10.1017/S0033291726103894 (PMC13234518; doi:10.1017/S0033291726103894)
Supplement: Huang et al. supplementary material [file S0033291726103894sup001.docx]

**Supplement S1:**

The following items from STAI loaded significantly onto the positive factor in the bifactor analysis of maternal mental health.

**Supplement Table S1.1: List of Positively Phrased Items and their loading onto the Positive Factor**

| **Question** | **Questionnaire and Question Number** | **Factor Loading** |
| --- | --- | --- |
| I feel pleasant (at this moment). | STAI20 | 0.64 |
| I feel steady (at this moment). | STAI19 | 0.60 |
| I feel content (at this moment). | STAI16 | 0.60 |
| I feel at ease (at this moment). | STAI5 | 0.59 |
| I feel secure (at this moment). | STAI2 | 0.59 |
| I feel self-confident (at this moment). | STAI11 | 0.58 |
| I am relaxed (at this moment). | STAI15 | 0.59 |
| I feel satisfied (at this moment). | STAI10 | 0.56 |
| I feel comfortable (at this moment). | STAI8 | 0.56 |
| I feel calm (at this moment). | STAI1 | 0.52 |
| I feel secure (generally). | STAI33 | 0.43 |
| I am content (generally). | STAI36 | 0.42 |
| I feel pleasant (generally). | STAI21 | 0.41 |
| I feel satisfied with myself (generally). | STAI23 | 0.39 |
| I am calm, cool, and collected (generally). | STAI27 | 0.36 |
| I feel rested (generally). | STAI26 | 0.30 |
| I am a steady person (generally). | STAI39 | 0.21 |

**Supplement S2: Scatterplots of Bayley’s Composite language score and BRIEF-2 GEC score against positive and negative maternal mental health**


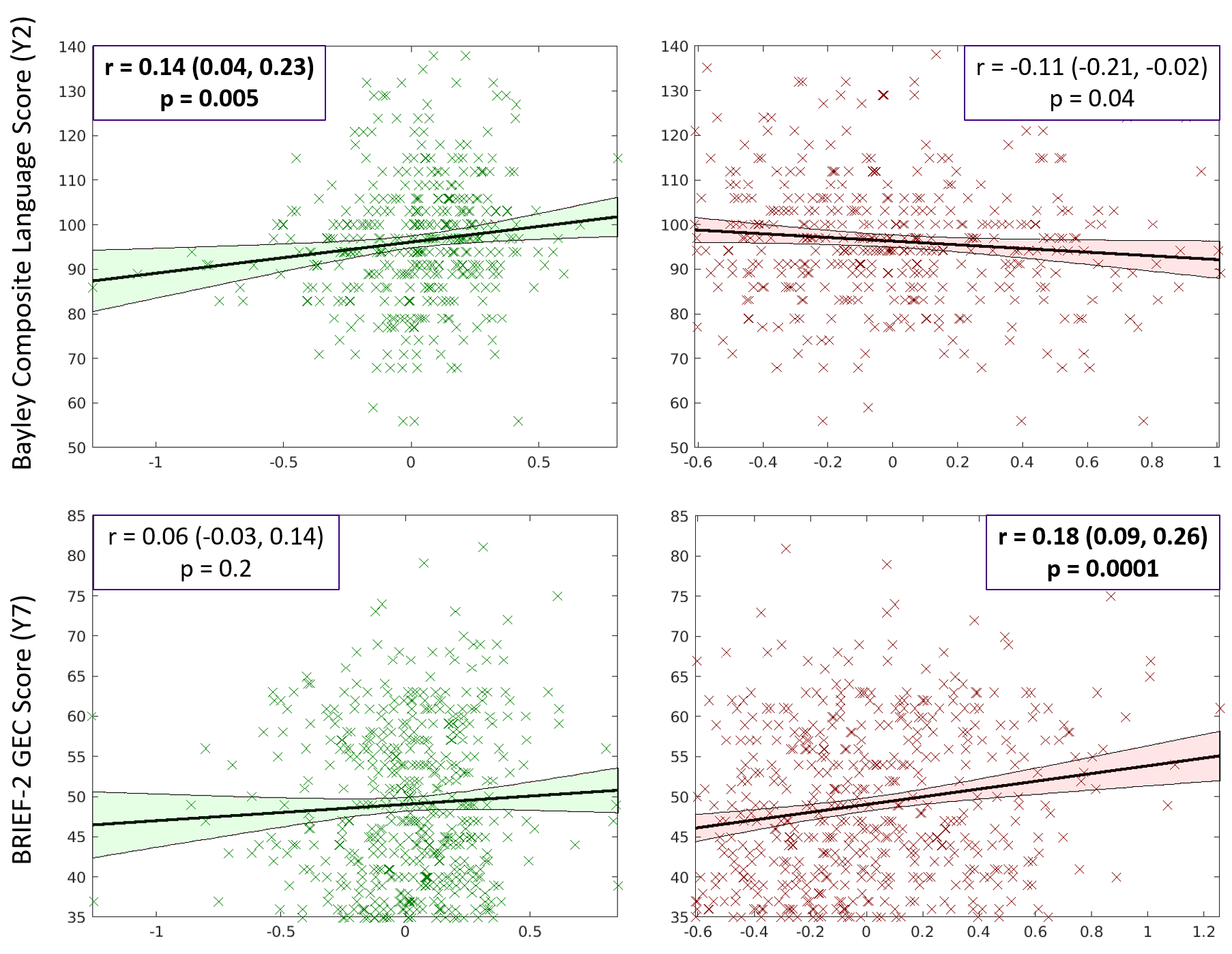


**Supplementary Figure S2.1:** Scatterplots showing the correlation positive and negative maternal mental health at Pregnancy Week 26 with Bayley composite language score at 2 years and BRIEF-2 total score at 7 years. Correlation values, 95% CI and p-values are included in the inserts. Bolded text indicates significant correlations after Bonferroni’s correction for multiple comparisons.

**Supplement S3: Partial Validation using an independent cohort (S-PRESTO)**

A partial validation of the study was carried out using the Singapore Preconception Study of Long-Term Maternal and Child Outcomes (S-PRESTO) cohort as an independent replication cohort. As the children in the SPRESTO study were all below 7 years old at the time of this study, the CDI-II was not administered and we were only able to test the correlations between positive and negative maternal mental health and the respective mediators. Due to limited overlap of measures used in both cohorts, we utilized slightly different measures of language and executive function. However, this could also be viewed as a strength as a replication would suggest the observed effects arise from the underlying language and executive function abilities and is not instrument dependent.

We applied the same bifactor structure to the three maternal mental health questionnaires obtained from the S-PRESTO mothers at pregnancy week 26 to extract out the equivalent general negative and positive maternal mental health factors. We used the Peabody Picture Vocabulary Test, 5th Ed (PPVT-5) as the measure of language ability. The Behaviour Rating Inventory of Executive Function, Preschool Version (BRIEF-P) was used as the measure of executive function. Both tests were administered during the same visit, with the child’s age at test between 4.5 to 6.3 years. Due to the wide range of ages, we utilized the standardized scores for both measures to adjust for age-related changes. The demographics table is tabulated in Supplement Table S3.1.

**Supplement Table S3.1:** **Summary of demographics for the S-PRESTO dataset and number of subjects, mean and standard deviation of the scores for all assessments.**

|  | **S-PRESTO cohort** | | |
| --- | --- | --- | --- |
|  | **n** | **mean** | **sd** |
| **Demographics** |  |  |  |
| Sex | 327 |  |  |
| - Male | 180 | (55.0) |  |
| - Female | 147 | (45.0) |  |
| Ethnicity | 327 |  |  |
| - Chinese | 255 | (78.0) |  |
| - Malay | 45 | (13.8) |  |
| - Indian | 22 | (6.7) |  |
| - Others/Mixed | 5 | (1.5) |  |
| Mother Highest Education | 315 |  |  |
| - Primary | 4 | (1.3) |  |
| - Secondary/Technical | 117 | (37.1) |  |
| - GCE ‘A’ levels/University | 194 | (61.6) |  |
| Monthly Household income (SGD) | 289 |  |  |
| - <1621 | 2 | (0.7) |  |
| - 1622-6144 | 78 | (27.0) |  |
| - >6145 | 209 | (72.3) |  |
| **Maternal Mental Health** |  |  |  |
| - Edinburgh Postnatal Depression Scale (PW26) | 301 | 6.48 | 4.51 |
| - Beck Depression Inventory 2 (PW26) | 297 | 8.92 | 6.44 |
| - State-Trait Anxiety Inventory (PW26) | 303 | 70.59 | 18.22 |
| - Negative Maternal Mental Health (PW26) | 297 | 0.01 | 0.48 |
| - Positive Maternal Mental Health (PW26) | 297 | 0.05 | 0.25 |
| **Offspring Measures (Y4.5-6.3)** |  |  |  |
| - Peabody Picture Vocabulary Test, 5^th^ Ed (Standard Score) | 205 | 96.73 | 15.08 |
| - Behavior Rating Inventory of Executive Function Preschool (Standard Score) | 197 | 95.90 | 19.35 |

***** Number in brackets indicate percentage of cohort. Note: Range for monthly household income is different due to different grading scheme used for the S-PRESTO cohort. Demographic variables were collected at recruitment. PW: Pregnancy Week. Y: Year

**Supplement S4: Estimates and confidence intervales for regression coefficients and mediation pathways for serial mediation analysis**

The full list of regression coefficients and mediation pathways are tabulated in Supplement Table S4.1 and S4.2. Significant estimates are bolded. We also included the estimates for covariates of no interest in Supplement Table S4.3 for completeness. As race was a categorical variable, we utilized Chinese as a base comparison as it was the majority category. The singular participant with Others race was grouped under Chinese to allow the model to converge.

Note: general_p26w: General (Negative) Maternal Mental Health Score at Prenatal Week 26, positive_p26w: Positive Maternal Mental Health Score at Prenatal Week 26, bayleys_CC_Y2: Bayley’s Scale of Infant Development Composite Language Score at 2 years, BRIEF_GEC_Y7: Behavior Rating Inventory of Executive Function 2, Global Executive Composite Score at 7 years, CDI_Total_Y9: Childhood Depression Inventory-II Total Score at 9 years.

**Supplement Table S4.1: Full list of regression estimates and confidence intervals for serial mediation analysis**

| **LHS** |  | **RHS** | **label** | **Estimate** | **S.E.** | **Lower CI** | **Upper CI** |
| --- | --- | --- | --- | --- | --- | --- | --- |
| bayleys_CC_Y2 | ~ | general_p26w | na1 | -0.034 | 0.055 | -0.141 | 0.080 |
| **bayleys_CC_Y2** | **~** | **positive_p26w** | **pa1** | **0.130** | **0.040** | **0.053** | **0.208** |
| **BRIEF_GEC_Y7** | **~** | **general_p26w** | **na2** | **0.223** | **0.047** | **0.127** | **0.314** |
| BRIEF_GEC_Y7 | ~ | positive_p26w | pa2 | 0.060 | 0.045 | -0.025 | 0.152 |
| BRIEF_GEC_Y7 | ~ | bayleys_CC_Y2 | d21 | 0.016 | 0.061 | -0.110 | 0.134 |
| CDI_Total_Y9 | ~ | general_p26w | ncp | -0.007 | 0.046 | -0.098 | 0.083 |
| CDI_Total_Y9 | ~ | positive_p26w | pcp | -0.040 | 0.045 | -0.127 | 0.051 |
| **CDI_Total_Y9** | **~** | **bayleys_CC_Y2** | **b1** | **-0.134** | **0.057** | **-0.244** | **-0.019** |
| **CDI_Total_Y9** | **~** | **BRIEF_GEC_Y7** | **b2** | **0.179** | **0.055** | **0.071** | **0.287** |

**Supplement Table S4.2: Full list of mediation pathways for serial mediation analysis**

| **Path** | **label** | **Estimate** | **S.E.** | **Lower CI** | **Upper CI** |
| --- | --- | --- | --- | --- | --- |
| general_p26w → bayleys_CC_Y2 → BRIEF_GEC_Y7 → CDI_Total_Y9 | na1*d21*b2 | 0.000 | 0.001 | -0.002 | 0.001 |
| general_p26w → bayleys_CC_Y2 → CDI_Total_Y9 | na1*b1 | 0.005 | 0.009 | -0.009 | 0.028 |
| **general_p26w → BRIEF_GEC_Y7 → CDI_Total_Y9** | **na2*b2** | **0.040** | **0.015** | **0.016** | **0.077** |
| positive_p26w → bayleys_CC_Y2 → BRIEF_GEC_Y7 → CDI_Total_Y9 | pa1*d21*b2 | 0.000 | 0.002 | -0.002 | 0.004 |
| **positive_p26w → bayleys_CC_Y2 → CDI_Total_Y9** | **pa1*b1** | **-0.017** | **0.010** | **-0.042** | **-0.003** |
| positive_p26w → BRIEF_GEC_Y7 → CDI_Total_Y9 | pa2*b2 | 0.011 | 0.009 | -0.003 | 0.036 |

**Supplement Table S4.3: Full list of covariate estimates and confidence intervals for serial mediation analysis**

| **LHS** |  | **RHS** | **Estimate** | **S.E.** | **Lower CI** | **Upper CI** |
| --- | --- | --- | --- | --- | --- | --- |
| **bayleys_CC_Y2** | **~** | **Monthly Household Income** | **0.192** | **0.086** | **0.029** | **0.365** |
| bayleys_CC_Y2 | **~** | Maternal Education Level | 0.097 | 0.062 | -0.027 | 0.215 |
| **bayleys_CC_Y2** | **~** | **Ethnicity (Malay)** | **0.299** | **0.129** | **0.048** | **0.556** |
| bayleys_CC_Y2 | ~ | Ethnicity (Indian) | 0.252 | 0.138 | -0.014 | 0.528 |
| **bayleys_CC_Y2** | **~** | **Sex** | **0.336** | **0.099** | **0.144** | **0.533** |
| BRIEF_GEC_Y7 | ~ | Monthly Household Income | -0.040 | 0.064 | -0.169 | 0.084 |
| **BRIEF_GEC_Y7** | **~** | **Maternal Education Level** | **0.104** | **0.050** | **0.007** | **0.202** |
| **BRIEF_GEC_Y7** |  | **Ethnicity (Malay)** | **-0.349** | **0.111** | **-0.565** | **-0.127** |
| **BRIEF_GEC_Y7** |  | **Ethnicity (Indian)** | **-0.299** | **0.128** | **-0.550** | **-0.043** |
| BRIEF_GEC_Y7 |  | Sex | -0.122 | 0.089 | -0.299 | 0.051 |
| CDI_Total_Y9 |  | Monthly Household Income | -0.037 | 0.066 | -0.167 | 0.093 |
| CDI_Total_Y9 |  | Maternal Education Level | -0.011 | 0.052 | -0.115 | 0.087 |
| **CDI_Total_Y9** |  | **Ethnicity (Malay)** | **0.251** | **0.114** | **0.025** | **0.475** |
| CDI_Total_Y9 |  | Ethnicity (Indian) | -0.207 | 0.130 | -0.455 | 0.058 |
| CDI_Total_Y9 | **~** | Sex | 0.103 | 0.089 | -0.071 | 0.277 |

**Supplement S5: Sensitivity analysis for serial mediation analysis**

To ensure the robustness and reliability of our findings, we performed three different sensitivity analysis. First, we ran the serial mediation model with only complete data and no imputation (Supplement S5.1). Second, we ran the serial mediation model with no covariates (Supplement S5.2). Lastly, we tested the sensitivity of the model to sample perturbation by re-estimating the significant pathways in randomly drawn subsamples comprising 10–90% of the full dataset (Supplement S5.3). This was repeated for 10 times at each sampling proportion.

Note: general_p26w: General (Negative) Maternal Mental Health Score at Prenatal Week 26, positive_p26w: Positive Maternal Mental Health Score at Prenatal Week 26, bayleys_CC_Y2: Bayley’s Scale of Infant Development Composite Language Score at 2 years, BRIEF_GEC_Y7: Behavior Rating Inventory of Executive Function 2, Global Executive Composite Score at 7 years, CDI_Total_Y9: Childhood Depression Inventory-II Total Score at 9 years.

**Supplement S5.1: Serial Mediation model with only complete data**

Using only participants with complete data for the 5 primary variables reduced the number of participants from 523 to 256. The indirect negative maternal mental health to child depressive symptoms pathway via executive function is still significant. The positive maternal mental health pathway remained similar in magnitude (β = -0.013) to the primary analysis (β = -0.017), but the confidence interval widened and marginally crossed zero (95% CI: −0.042 to 0.001). This attenuation in statistical significance likely reflects the substantially reduced sample size and corresponding loss of statistical power rather than a meaningful change in the estimated effect.

**Supplement Table S5.1.1: Full list of regression estimates and confidence intervals for serial mediation analysis with only complete data**

| **LHS** |  | **RHS** | **label** | **Estimate** | **S.E.** | **Lower CI** | **Upper CI** |
| --- | --- | --- | --- | --- | --- | --- | --- |
| bayleys_CC_Y2 | ~ | general_p26w | na1 | -0.006 | 0.061 | -0.127 | 0.114 |
| **bayleys_CC_Y2** | **~** | **positive_p26w** | **pa1** | **0.125** | **0.054** | **0.019** | **0.232** |
| **BRIEF_GEC_Y7** | **~** | **general_p26w** | **na2** | **0.169** | **0.065** | **0.038** | **0.291** |
| BRIEF_GEC_Y7 | ~ | positive_p26w | pa2 | 0.053 | 0.068 | -0.075 | 0.193 |
| BRIEF_GEC_Y7 | ~ | bayleys_CC_Y2 | d21 | 0.035 | 0.070 | -0.104 | 0.170 |
| CDI_Total_Y9 | ~ | general_p26w | ncp | -0.098 | 0.062 | -0.219 | 0.025 |
| CDI_Total_Y9 | ~ | positive_p26w | pcp | -0.090 | 0.077 | -0.243 | 0.056 |
| CDI_Total_Y9 | ~ | bayleys_CC_Y2 | b1 | -0.101 | 0.062 | -0.225 | 0.019 |
| **CDI_Total_Y9** | **~** | **BRIEF_GEC_Y7** | **b2** | **0.206** | **0.071** | **0.067** | **0.346** |

**Supplement Table S5.1.2: Full list of mediation pathways for serial mediation analysis with only complete data**

| **Path** | **label** | **Estimate** | **S.E.** | **Lower CI** | **Upper CI** |
| --- | --- | --- | --- | --- | --- |
| general_p26w → bayleys_CC_Y2 → BRIEF_GEC_Y7 → CDI_Total_Y9 | na1*d21*b2 | 0.000 | 0.001 | -0.003 | 0.002 |
| general_p26w → bayleys_CC_Y2 → CDI_Total_Y9 | na1*b1 | 0.001 | 0.007 | -0.012 | 0.020 |
| **general_p26w → BRIEF_GEC_Y7 → CDI_Total_Y9** | **na2*b2** | **0.035** | **0.018** | **0.008** | **0.084** |
| positive_p26w → bayleys_CC_Y2 → BRIEF_GEC_Y7 → CDI_Total_Y9 | pa1*d21*b2 | 0.001 | 0.002 | -0.002 | 0.007 |
| positive_p26w → bayleys_CC_Y2 → CDI_Total_Y9 | pa1*b1 | -0.013 | 0.010 | -0.042 | 0.001 |
| positive_p26w → BRIEF_GEC_Y7 → CDI_Total_Y9 | pa2*b2 | 0.011 | 0.016 | -0.012 | 0.053 |

**Supplement Table S5.1.3: Full list of covariate estimates and confidence intervals for serial mediation analysis with only complete data**

| **LHS** |  | **RHS** | **Estimate** | **S.E.** | **Lower CI** | **Upper CI** |
| --- | --- | --- | --- | --- | --- | --- |
| **bayleys_CC_Y2** | **~** | **Monthly Household Income** | **0.273** | **0.095** | **0.093** | **0.463** |
| bayleys_CC_Y2 | **~** | Maternal Education Level | 0.086 | 0.064 | -0.042 | 0.209 |
| **bayleys_CC_Y2** | **~** | **Ethnicity (Malay)** | **0.415** | **0.152** | **0.116** | **0.717** |
| **bayleys_CC_Y2** | **~** | **Ethnicity (Indian)** | **0.448** | **0.167** | **0.123** | **0.786** |
| **bayleys_CC_Y2** | **~** | **Sex** | **0.267** | **0.118** | **0.040** | **0.500** |
| BRIEF_GEC_Y7 | ~ | Monthly Household Income | -0.094 | 0.092 | -0.265 | 0.096 |
| BRIEF_GEC_Y7 | ~ | Maternal Education Level | 0.130 | 0.073 | -0.012 | 0.273 |
| **BRIEF_GEC_Y7** |  | **Ethnicity (Malay)** | **-0.395** | **0.161** | **-0.708** | **-0.074** |
| **BRIEF_GEC_Y7** |  | **Ethnicity (Indian)** | **-0.400** | **0.188** | **-0.771** | **-0.029** |
| **BRIEF_GEC_Y7** |  | **Sex** | **-0.277** | **0.125** | **-0.520** | **-0.028** |
| CDI_Total_Y9 |  | Monthly Household Income | -0.077 | 0.100 | -0.272 | 0.124 |
| CDI_Total_Y9 |  | Maternal Education Level | -0.076 | 0.073 | -0.222 | 0.064 |
| CDI_Total_Y9 |  | Ethnicity (Malay) | 0.153 | 0.173 | -0.179 | 0.497 |
| **CDI_Total_Y9** |  | **Ethnicity (Indian)** | **-0.429** | **0.184** | **-0.781** | **-0.058** |
| CDI_Total_Y9 | **~** | Sex | 0.224 | 0.128 | -0.014 | 0.490 |

**Supplement S5.2: Serial Mediation model with no covariates**

We also executed the mediation model with no covariates to demonstrate that the significant pathways were independent of the covariate effects. Both pathways remain significant in the absence of covariates.

**Supplement Table S5.2.1: Full list of regression estimates and confidence intervals for serial mediation analysis with no covariates**

| **LHS** |  | **RHS** | **label** | **Estimate** | **S.E.** | **Lower CI** | **Upper CI** |
| --- | --- | --- | --- | --- | --- | --- | --- |
| bayleys_CC_Y2 | ~ | general_p26w | na1 | -0.097 | 0.058 | -0.209 | 0.023 |
| **bayleys_CC_Y2** | **~** | **positive_p26w** | **pa1** | **0.117** | **0.041** | **0.037** | **0.198** |
| **BRIEF_GEC_Y7** | **~** | **general_p26w** | **na2** | **0.176** | **0.046** | **0.082** | **0.264** |
| BRIEF_GEC_Y7 | ~ | positive_p26w | pa2 | 0.057 | 0.046 | -0.028 | 0.150 |
| BRIEF_GEC_Y7 | ~ | bayleys_CC_Y2 | d21 | 0.016 | 0.059 | -0.103 | 0.131 |
| CDI_Total_Y9 | ~ | general_p26w | ncp | 0.023 | 0.044 | -0.063 | 0.109 |
| CDI_Total_Y9 | ~ | positive_p26w | pcp | -0.029 | 0.044 | -0.113 | 0.060 |
| **CDI_Total_Y9** | **~** | **bayleys_CC_Y2** | **b1** | **-0.149** | **0.056** | **-0.261** | **-0.042** |
| **CDI_Total_Y9** | **~** | **BRIEF_GEC_Y7** | **b2** | **0.161** | **0.054** | **0.058** | **0.267** |

**Supplement Table S5.2.2: Full list of mediation pathways for serial mediation analysis with no covariates**

| **Path** | **label** | **Estimate** | **S.E.** | **Lower CI** | **Upper CI** |
| --- | --- | --- | --- | --- | --- |
| general_p26w → bayleys_CC_Y2 → BRIEF_GEC_Y7 → CDI_Total_Y9 | na1*d21*b2 | 0.000 | 0.001 | -0.003 | 0.001 |
| general_p26w → bayleys_CC_Y2 → CDI_Total_Y9 | na1*b1 | 0.014 | 0.011 | -0.001 | 0.045 |
| **general_p26w → BRIEF_GEC_Y7 → CDI_Total_Y9** | **na2*b2** | **0.028** | **0.012** | **0.010** | **0.060** |
| positive_p26w → bayleys_CC_Y2 → BRIEF_GEC_Y7 → CDI_Total_Y9 | pa1*d21*b2 | 0.000 | 0.001 | -0.002 | 0.004 |
| **positive_p26w → bayleys_CC_Y2 → CDI_Total_Y9** | **pa1*b1** | **-0.017** | **0.010** | **-0.043** | **-0.003** |
| positive_p26w → BRIEF_GEC_Y7 → CDI_Total_Y9 | pa2*b2 | 0.009 | 0.009 | -0.003 | 0.033 |

**Supplement S5.3: Repeated analysis with population subsampling**

To assess the sensitivity of the structural equation model to sample size, we conducted a subsampling analysis. Random subsets of the full dataset were generated at 10% increments from 10% to 90% of the total sample. For each subsample proportion, 10 independent random draws without replacement were performed. The SEM model was re-estimated for each subsample using the same model specification as in the primary analysis. We then evaluated the consistency of the two signfiicant pathway estimate across subsamples, including the proportion of iterations in which the pathway remained statistically significant. This approach provides an empirical assessment of the stability of model estimates under reduced sample sizes and helps determine whether observed effects are robust to perturbations in sample composition.

The indirect positive maternal mental health pathway (Supplement Table S5.3.1) remained consistently detectable at higher subsample proportions. The pathway was statistically significant in all 10 iterations at 90% subsampling while 8 out of 10 iterations were significant at 80% of the full sample size. For the indirect negative maternal mental health pathway (Supplement Table S5.3.2), the pathway was statistically significant for all 10 iterations at 60 to 90% subsampling and 9 out of 10 iterations were significant at 50% subsampling.

For both pathways, the proportion of iterations with statistical significance declined with reducing subsample sizes, consistent with reduced statistical power at smaller sample sizes. Importantly, the direction and magnitude of both pathway estimates (β≈-0.02 for positive maternal mental health and β≈0.04 for negative maternal mental health) remained comparable across subsamples, indicating that the observed pathway effects is relatively stable.

**Supplement Table S5.3.1: Estimates for the indirect positive maternal mental health** **→ child language** → **child depressive symptoms pathways for different subsamples.**

|  | **Subsample Percentage (%)** | | | | | | | | |
| --- | --- | --- | --- | --- | --- | --- | --- | --- | --- |
| Iteration | **90** | **80** | **70** | **60** | **50** | **40** | **30** | **20** | **10** |
| 1 | **-0.020 (-0.048, -0.004)** | -0.018 (-0.046, 0.001) | **-0.019 (-0.056, -0.001)** | -0.013 (-0.049, 0.002) | -0.035 (-0.089, 0.001) | **-0.029 (-0.084, -0.001)** | -0.027 (-0.103, 0.004) | -0.013 (-0.133, 0.020) | -0.043 (-0.535, 0.125) |
| 2 | **-0.021 (-0.050, -0.004)** | **-0.024 (-0.055, -0.005)** | -0.017 (-0.048, 0.001) | **-0.032 (-0.078, -0.004)** | -0.009 (-0.044, 0.004) | -0.028 (-0.080, 0.001) | -0.046 (-0.144, 0.008) | 0.008 (-0.021, 0.093) | -0.035 (-1.338, 0.346) |
| 3 | **-0.019 (-0.045, -0.004)** | **-0.015 (-0.041, -0.001)** | -0.019 (-0.055, 0.001) | **-0.021 (-0.053, -0.003)** | -0.021 (-0.066, 0.002) | -0.007 (-0.042, 0.014) | -0.003 (-0.053, 0.013) | -0.018 (-0.149, 0.102) | -0.020 (-0.425, 0.303) |
| 4 | **-0.016 (-0.044, -0.001)** | **-0.017 (-0.044, -0.002)** | **-0.023 (-0.059, -0.004)** | -0.016 (-0.046, 0.004) | -0.014 (-0.046, 0.001) | -0.011 (-0.063, 0.008) | -0.030 (-0.112, 0.009) | -0.042 (-0.204, 0.027) | -0.012 (-0.268, 0.208) |
| 5 | **-0.018 (-0.043, -0.002)** | **-0.020 (-0.049, -0.003)** | **-0.023 (-0.055, -0.004)** | -0.017 (-0.054, 0.000) | -0.014 (-0.051, 0.011) | -0.001 (-0.035, 0.020) | -0.018 (-0.107, 0.020) | -0.009 (-0.093, 0.022) | -0.001 (-0.353, 0.219) |
| 6 | **-0.013 (-0.038, 0.000)** | **-0.012 (-0.039, -0.001)** | **-0.026 (-0.060, -0.006)** | -0.017 (-0.056, 0.000) | **-0.021 (-0.059, -0.001)** | -0.024 (-0.075, 0.000) | -0.020 (-0.084, 0.007) | 0.020 (-0.016, 0.146) | -0.012 (-0.645, 0.242) |
| 7 | **-0.015 (-0.043, -0.001)** | **-0.026 (-0.057, -0.006)** | -0.011 (-0.040, 0.001) | -0.013 (-0.050, 0.008) | **-0.022 (-0.061, -0.002)** | -0.040 (-0.117, 0.001) | -0.024 (-0.099, 0.003) | 0.002 (-0.023, 0.050) | 0.003 (-0.213, 0.337) |
| 8 | **-0.015 (-0.040, -0.002)** | -0.010 (-0.035, 0.001) | -0.017 (-0.047, 0.000) | **-0.030 (-0.074, -0.002)** | **-0.036 (-0.083, -0.008)** | -0.030 (-0.101, 0.005) | -0.039 (-0.134, 0.014) | -0.016 (-0.097, 0.017) | 0.009 (-0.483, 0.457) |
| 9 | **-0.017 (-0.045, -0.002)** | **-0.025 (-0.055, -0.006)** | -0.010 (-0.035, 0.001) | **-0.022 (-0.064, -0.001)** | -0.006 (-0.036, 0.006) | -0.006 (-0.050, 0.008) | -0.028 (-0.088, 0.011) | -0.011 (-0.166, 0.086) | -0.004 (-0.239, 0.092) |
| 10 | **-0.020 (-0.048, -0.004)** | **-0.021 (-0.052, -0.003)** | -0.010 (-0.038, 0.004) | **-0.031 (-0.075, -0.004)** | -0.004 (-0.037, 0.008) | -0.014 (-0.059, 0.004) | -0.030 (-0.085, 0.000) | -0.030 (-0.144, 0.017) | -0.144 (-0.581, 0.249) |
| *Average* | *-0.017 (-0.044, -0.002)* | *-0.019 (-0.047, -0.003)* | *-0.018 (-0.049, -0.001)* | *-0.021 (-0.060, 0.000)* | *-0.018 (-0.057, 0.002)* | *-0.019 (-0.071, 0.006)* | *-0.027 (-0.101, 0.009)* | *-0.011 (-0.105, 0.058)* | *-0.026 (-0.508, 0.258)* |

*Bold text indicates significant pathways. Average values are not bolded since they were averaged across 10 different iterations.

**Supplement Table S5.3.2: Estimates for the indirect negative maternal mental health** **→ child executive function** → **child depressive symptoms pathways for different subsamples.**

|  | **Subsample Percentage (%)** | | | | | | | | |
| --- | --- | --- | --- | --- | --- | --- | --- | --- | --- |
| Iteration | **90** | **80** | **70** | **60** | **50** | **40** | **30** | **20** | **10** |
| 1 | **0.039 (0.014, 0.079)** | **0.024 (0.002, 0.058)** | **0.065 (0.029, 0.119)** | **0.029 (0.002, 0.072)** | **0.038 (0.000, 0.099)** | **0.055 (0.005, 0.145)** | **0.059 (0.011, 0.143)** | 0.065 (-0.039, 0.207) | 0.006 (-0.091, 0.181) |
| 2 | **0.043 (0.017, 0.083)** | **0.032 (0.007, 0.072)** | **0.023 (0.004, 0.063)** | **0.036 (0.003, 0.089)** | 0.030 (-0.007, 0.08) | 0.020 (-0.008, 0.072) | -0.004 (-0.06, 0.044) | 0.023 (-0.020, 0.131) | 0.094 (-0.140, 1.075) |
| 3 | **0.038 (0.014, 0.076)** | **0.042 (0.016, 0.085)** | **0.047 (0.017, 0.092)** | **0.040 (0.007, 0.09)** | **0.037 (0.009, 0.085)** | **0.035 (0.006, 0.095)** | 0.016 (-0.024, 0.084) | 0.092 (-0.005, 0.297) | -0.033 (-0.413, 0.088) |
| 4 | **0.032 (0.009, 0.069)** | **0.043 (0.015, 0.087)** | **0.042 (0.010, 0.088)** | **0.042 (0.013, 0.093)** | **0.028 (0.002, 0.078)** | 0.033 (-0.006, 0.107) | 0.055 (-0.004, 0.165) | 0.009 (-0.033, 0.097) | 0.125 (-0.015, 0.38) |
| 5 | **0.033 (0.010, 0.07)** | **0.041 (0.013, 0.083)** | **0.028 (0.006, 0.07)** | **0.048 (0.013, 0.109)** | **0.054 (0.008, 0.122)** | 0.041 (-0.003, 0.111) | 0.021 (-0.003, 0.112) | **0.076 (0.003, 0.222)** | -0.014 (-0.217, 0.063) |
| 6 | **0.035 (0.010, 0.073)** | **0.041 (0.014, 0.083)** | **0.020 (0.001, 0.055)** | **0.045 (0.015, 0.097)** | **0.034 (0.006, 0.088)** | 0.030 (-0.014, 0.092) | 0.016 (-0.020, 0.084) | 0.018 (-0.033, 0.139) | -0.033 (-0.618, 0.14) |
| 7 | **0.041 (0.015, 0.08)** | **0.044 (0.015, 0.086)** | **0.034 (0.008, 0.081)** | **0.040 (0.006, 0.091)** | **0.057 (0.016, 0.12)** | 0.028 (-0.003, 0.106) | **0.055 (0.003, 0.159)** | **0.037 (0.001, 0.113)** | 0.018 (-0.057, 0.215) |
| 8 | **0.044 (0.017, 0.085)** | **0.043 (0.013, 0.091)** | **0.042 (0.011, 0.091)** | **0.045 (0.014, 0.098)** | **0.041 (0.006, 0.098)** | **0.065 (0.018, 0.142)** | 0.040 (-0.003, 0.121) | 0.033 (-0.010, 0.115) | 0.015 (-0.089, 0.613) |
| 9 | **0.038 (0.013, 0.078)** | **0.036 (0.011, 0.073)** | **0.038 (0.010, 0.087)** | **0.047 (0.014, 0.107)** | **0.040 (0.006, 0.106)** | 0.008 (-0.035, 0.062) | **0.060 (0.012, 0.158)** | 0.031 (-0.007, 0.13) | -0.014 (-0.209, 0.033) |
| 10 | **0.039 (0.015, 0.078)** | **0.039 (0.014, 0.08)** | **0.032 (0.008, 0.07)** | **0.037 (0.008, 0.085)** | **0.027 (0.002, 0.081)** | 0.030 (-0.002, 0.092) | **0.045 (0.005, 0.142)** | -0.009 (-0.088, 0.012) | 0.011 (-0.201, 0.400) |
| *Average* | *0.038 (0.013, 0.077)* | *0.039 (0.012, 0.080)* | *0.037 (0.010, 0.082)* | *0.041 (0.010, 0.093)* | *0.039 (0.005, 0.096)* | *0.035 (-0.004, 0.102)* | *0.036 (-0.008, 0.121)* | *0.038 (-0.023, 0.146)* | *0.018 (-0.205, 0.319)* |

*Bold text indicates significant pathways. Average values are not bolded since they were averaged across 10 different iterations.
